# Direct evidence for α ether linkage between lignin and carbohydrates in wood cell walls

**DOI:** 10.1038/s41598-018-24328-9

**Published:** 2018-04-25

**Authors:** Hiroshi Nishimura, Akihiro Kamiya, Takashi Nagata, Masato Katahira, Takashi Watanabe

**Affiliations:** 1 Research Institute for Sustainable Humanosphere (RISH), Kyoto University, Uji, 611-0011 Japan; 2 Institute of Advanced Energy (IAE), Kyoto University, Uji, 611-0011 Japan

## Abstract

Cross-linking between lignin and polysaccharide in plant cell-wall determines physical, chemical, and biological features of lignocellulosic biomass. Since Erdmann’s first report in 1866, numerous studies have suggested the presence of a bond between hemicelluloses and lignin; however, no clear evidence for this interaction has been reported. We describe the first direct proof of covalent bonding between plant cell-wall polysaccharides and lignin. Nuclear magnetic resonance spectroscopy was used to observe the long-range correlations through an α-ether bond between lignin and the primary hydroxyl group of a mannose residue in glucomannan. Complete signal assignment of the cognate structural units was also achieved. Thus, we identified lignin–carbohydrate bonds by complete connectivity analysis from the phenylpropane unit to the carbohydrate moiety.

## Introduction

**Figure 1:**
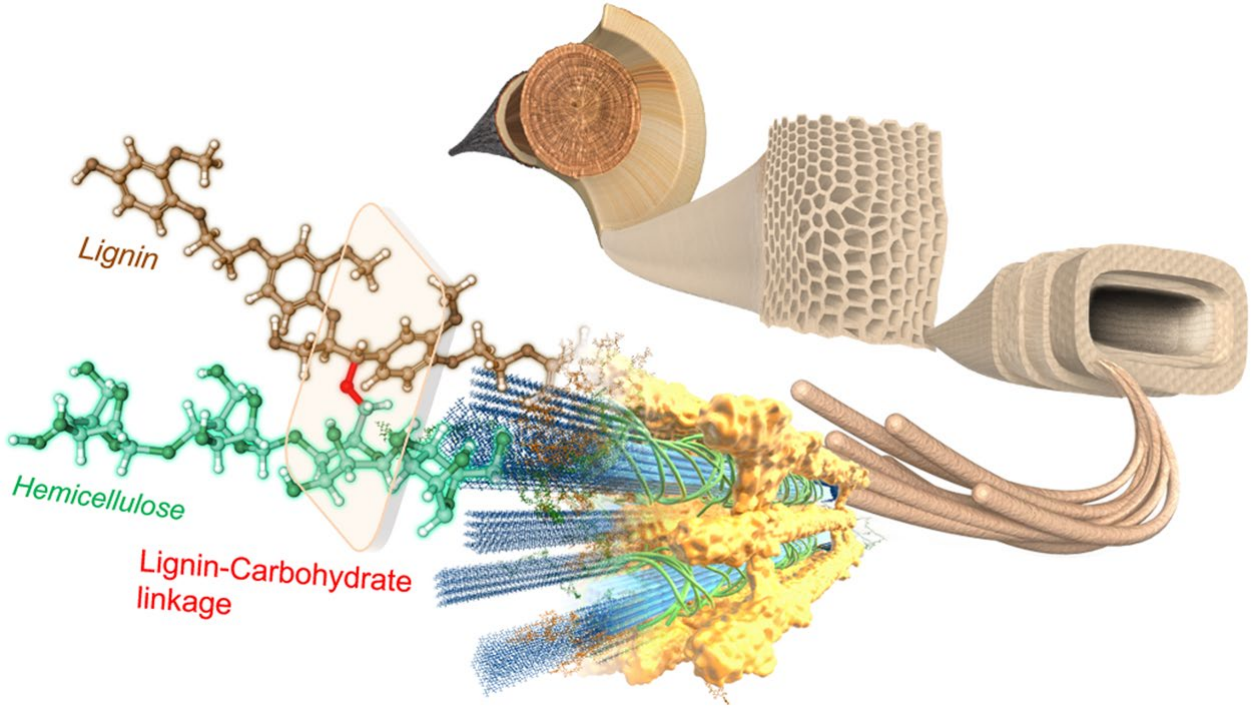
A three-dimensional view of the lignin–carbohydrate complex (LCC) in the wood cell wall. An illustration of wood cell walls and their ultrastructure focusing on the ether linkage between lignin and carbohydrate, a structural feature that has remained unresolved for more than 150 years, and whose presence and location is elucidated in this study. Wood fibre networks consist of wood cell walls composed of cellulose, hemicellulose and lignin, the most abundant organic resources on earth. These polymer combinations give the plant cell wall strength and structure; however, the cell wall’s robustness is a barrier for the separation and utilisation of plant-based resources. Thus, the LCC is a key structure to elucidate physiologically, physically and industrially.

Lignin is a key adaptation to terrestrial life that reinforces plant cell walls; this compound forms the hydrophobic xylem vessels that transport water, and it supports plant bodies by co-localising with polysaccharides, cellulose, and hemicellulose. Lignin is thought to associate with hemicelluloses through covalent and non-covalent bonding. The complex thus assembled is called the lignin–carbohydrate complex (LCC, Fig. 1).

Recently, plant biomass has been highlighted as an alternative carbon source to fossil fuels. Plant cell walls, naturally designed to be durable, have good potential to become precursors for a variety of chemicals, fuels and value-added products; however, efficient separation of plant biomass is still challenging. Thus, a deeper fundamental understanding of the characteristics of plant cell walls, in particular with regard to the nature of the linkage between lignin and polysaccharides, is of great importance.

The most difficult process in biomass conversion is the separation of lignin from polysaccharides; however, LCCs are highly structurally rigid complexes, and separation of these polymers is difficult^1,2^. In 1866, Erdman^3^ hypothesised that lignin and polysaccharides combine chemically with each other to form a ‘glycolignose’. Subsequently, numerous investigations have been conducted to elucidate the structure and nature of LCCs in plant cell walls; however, no direct proof of the presence of chemical bonds between lignin and polysaccharides has been reported. To improve our understanding of plant cell wall structure and to develop efficient separation techniques to be implemented in lignocellulosic biorefineries, additional information on the chemical linkages between lignin and carbohydrates is necessary.

Since Björkman first developed procedures for fractionating LCCs from ball-milled wood^4,5^, several additional LCC fractionation methods have been proposed^1,6,7^, and the structure of LCC has been further analysed. Historically, the presence and nature of covalent bonds between lignin and carbohydrates has been investigated by indirect methods, such as alkaline degradation, Smith degradation, methylation analysis and IR and 1D NMR spectroscopies. Four types of lignin–carbohydrate (LC) linkages have been proposed to exist. Specifically, α ether linkages have been reported as the major LC bonds in LCCs^8–13^, with other LC bonds, such as α ester^9,12–16^, phenyl glycoside^15,17,18^ and acetal bonds^19^ also being reported. More recently, 2D ^1^H–^13^C heteronuclear single-quantum correlation (HSQC) NMR spectroscopy has been applied to the analysis of LC bonds, and the presence of α ether and phenyl glycoside bonds has been reported^20–24^. In addition, γ-ester LC linkages, instead of α esters, have been reported^22,23^. These observations and assignments were based on ^1^H–^13^C HSQC correlations. However, the chemical shifts of the relevant signals are not exactly identical to those of synthetic model compounds. Furthermore, the possibility of mis-assignment of the signals due to severe overlapping of the signals from other unidentified structures cannot be excluded.

In the present study, we conducted long-range correlation NMR experiments to obtain direct evidence on the chemical linkages between the phenylpropane units of lignin and hemicelluloses. To observe the LC bonds by long-range correlation NMR, condensation of the LC bonds in isolated LCC fractions is crucial; the frequency of LC bonds in LCCs isolated from plant cell walls is too low for NMR experiments to provide information about them. In order to prepare LCC fragments possessing a number of LC bonds high enough to suit NMR sensitivity, we concentrated the LC bonds in water-soluble neutral LCCs from *Pinus densiflora* (Japanese red pine) wood by carrying out the enzymatic digestion of the sugar moiety and using a polyvinyl gel characterised by an affinity for lignin and size-exclusion activity^25,26^.

Herein, we focus on the analysis of ether linkages among native LC bonds. During the polymerisation of monolignols, quinone methides are formed by coupling β- and phenoxy- radicals. These quinone methides then react with hydroxyl groups of the carbohydrates to form α ether bonds between lignin and carbohydrates. In previous studies, we demonstrated that primary hydroxyl groups (C6 position) of hexoses preferentially participate in α ether linkages through 2,3-dichloro-5,6-dicyanobenzoquinone (DDQ) oxidation followed by Prehm’s methylation^12,26^. Due to selective ether cleavage by DDQ at the α position and the labelling of the cleavage points of carbohydrates under neutral conditions, this result strongly supports the existence of an α ether bond. To obtain further unequivocal direct evidence of the presence of this bond, we used a combination of ^1^H–^13^C HSQC, heteronuclear multiple bond correlation (HMBC) and ^13^C-edited total correlation NMR spectroscopy (TOCSY) on the LC-bond-concentrated LCC fraction and demonstrated that in it the C-6 position of mannose was chemically linked to the α position of lignin.

## Results

### Preparation of the neutral LCC fraction by extraction and anion-exchange chromatography

**Figure 2:**
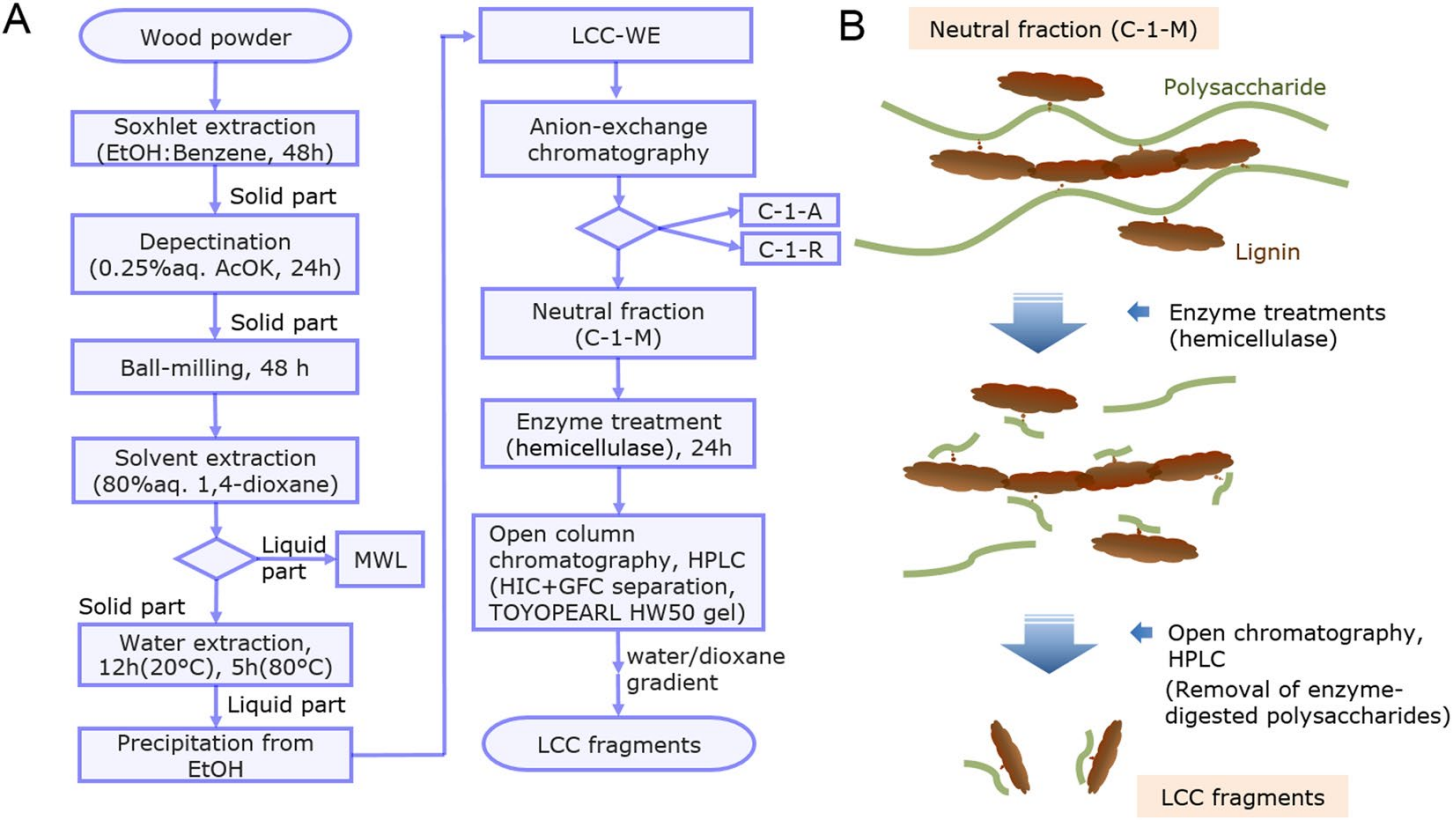
Fractionation flowchart (**A**) and schematic depiction of the procedure (**B**) for purifying LCC fragments from wood powder through a combination of enzymatic digestion, gel filtration and hydrophobic interaction chromatographic purification using TOYOPEARL HW 50 gel for removing digested polysaccharides^25,26^.

LCC is a composite polymer comprising lignin and polysaccharides. To obtain original LCC from wood cell walls, we have focused on the MWL residue. MWL mostly consists of lignin, and its residue is in large part composed of polysaccharides, cellulose, and hemicellulose, but it also contains LCC. Here we isolated a water-soluble LCC fraction (LCC-WE) from the residue of MWL extracts. A flowchart of the LCC fractionation procedure is reported in Fig. 2A. To classify hemicellulose part, LCC-WE was separated into its neutral (C-1-M) and acidic (C-1-A) fractions as described previously^27^. C-1-M is a water-soluble LCC fraction containing structures in which pendant-like lignin is attached to a hemicellulose chain. In normal pine wood, this fraction is composed of lignin bound to chains of pure acetyl glucomannan. In reaction wood, β-1,4-galactan is present in this fraction, but arabino-glucuronoxylan is not. Sugar analysis showed that the neutral sugar content in C-1-M was 96.2%. The sugar composition was Rha 0.6%, Ara 1.2%, Xyl 0.3%, Man 59.0%, Gal 16.3% and Glc 22.6%. Smith degradation of C-1-M yielded 1.7% ethylene glycol, 4.9% glycerol, 81.0% erythritol, 8.3% mannose and 4.1% glucose. Xylose and galactose were not detected by this approach, supporting the hypothesis that the major component in C-1-M is partially acetylated glucomannan and that β-1,4-linked galactan is also present in the fraction.

### Preparation of pure LCC fragments through a combination of enzymatic degradation and hydrophobic-gel chromatography

A high concentration of LC bonds and their high purity are crucial prerequisites for the complete structural elucidation of LCC. Thus, enzymatic hydrolysis of C-1-M was conducted. *Cellulosin* GM5, an enzyme with mainly mannanase activity, was allowed to react with C-1-M. After this digestion, LCC fragments were separated from the digested saccharides using a polyvinyl gel open column with an affinity for lignin. The LCC fragment was then purified by HPLC with a handmade-column filled with the same gel. This separation is a hybrid procedure consisting of gel filtration chromatography (GFC) and hydrophobic interaction chromatography (HIC)^25,26^. A flowchart of this procedure and a schematic depiction of its stages are reported in Fig. 2A,B, respectively.

### Structural elucidation of neutral LCC using long-range-correlation 2D- and 3D-NMR

**Figure 3:**
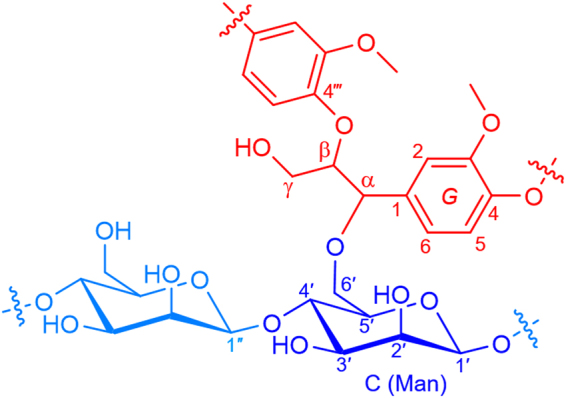
The partial chemical structure of α-ether-type LCC, whose characteristics were elucidated in this study. The C-6′ position of β (1″ → 4′)-linked D-mannose units in glucomannan is linked to the α position of the β-*O*-4′″ lignin subunits through an ether bond.

**Figure 4:**
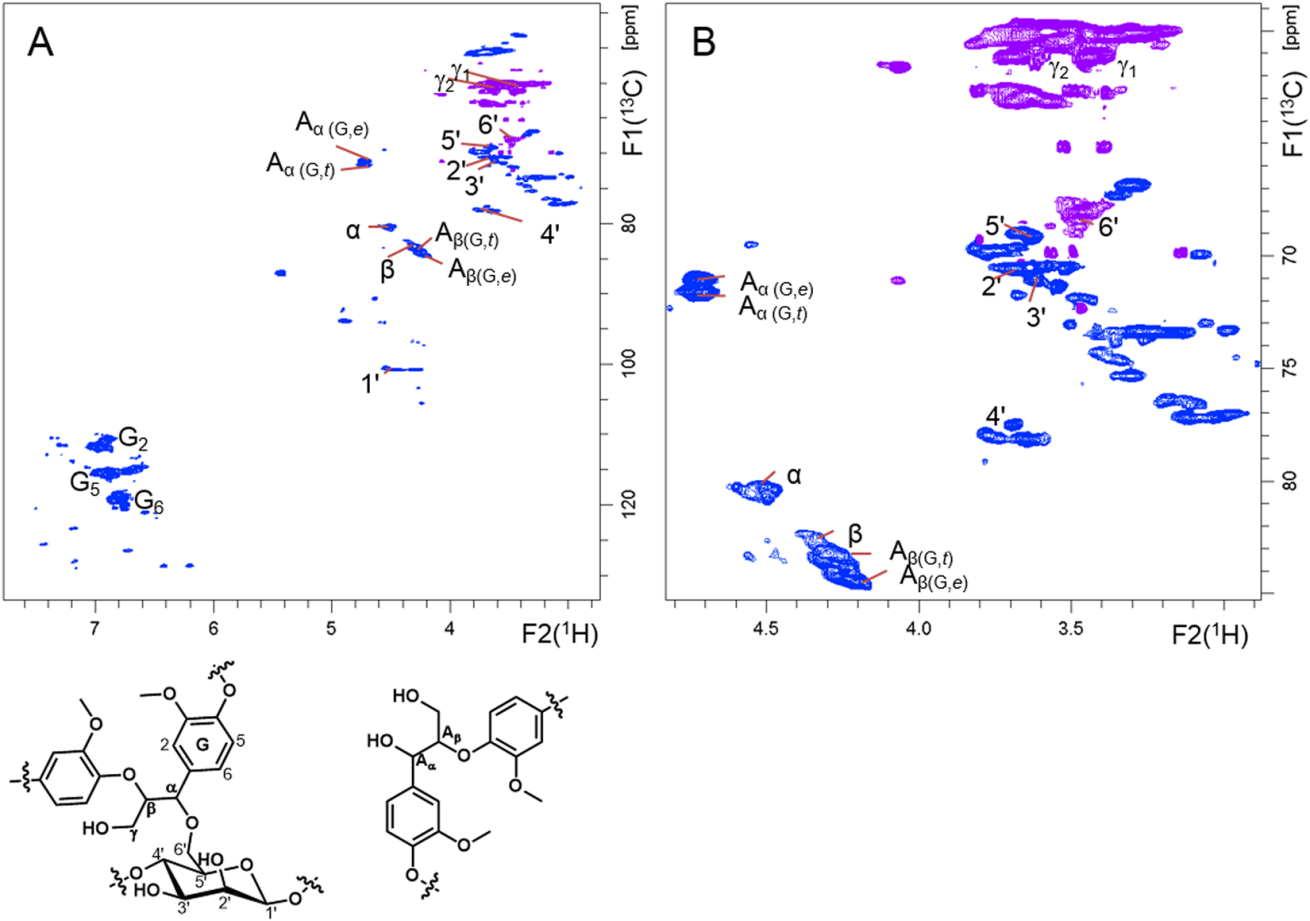
The multiplicity edited ^1^H–^13^C HSQC NMR spectra of the α-ether-type LCC: overall (**A**) and magnified (**B**) spectrum. Signals originating from LCC α-ether linkages are labelled as the partial chemical structure on this figure, also shown in Fig. 3.‘α’, ‘β’ and ‘γ’ are ^1^H–^13^C correlations at the corresponding positions of lignin moiety of LCC. ‘1′– 6′’ are carbohydrate moiety (β-1,4-D-mannose) of LCC. Free β-*O*-4′″ lignin units, uncombined with carbohydrates, are abbreviated as ‘A’. Guaiacyl lignin units, *erythro* form, and *threo* form are abbreviated as ‘G’, ‘*e*’ and ‘*t*’, respectively. Signals originating from CH and CH_3_ are reported in blue. Methylene CH_2_ signals are reported in purple.

We analysed the purified LCC fraction using NMR spectroscopy. In Fig. 3 is reported the proposed chemical structure of the α-ether-type LCC. In the ^1^H–^13^C HSQC NMR spectrum, a Cα–Hα correlation signal corresponding to the α-ether-type LCC was observed at δ_H_/δ_C_ = 4.50 ppm/80.2 ppm, as can be evinced from Fig. 4A,B, in accordance with the values observed for LCC model compounds^28,29^. In previous studies on LCCs, signals were assigned based only on a pair of chemical shifts originating from one-bond spin couplings between ^1^H and ^13^C^22,23^. However, these results do not conclusively prove the presence of covalent bonds between lignin and saccharides. In the present study, through-bond correlations with 2- to 4-bond spin couplings were analysed by ^1^H–^13^C HMBC, 2D HSQC-TOCSY, and 3D TOCSY-HSQC measurements. Correlations of the sugar moiety of LCC partly overlapped in the 2D HSQC-TOCSY; thus, more definite assignments were achieved by extending the correlation analysis into the third dimension employing 3D TOCSY-HSQC.

We then succeeded in the identification of α ether LC bonds. We directly observed the correlation signals associated with α ether LC bonds between lignin-α and the primary hydroxyl group of the mannose residue, C-6′. We were also able to assign all signals related to the LCC structural units by complete connectivity analysis, from the phenylpropane unit to the carbohydrate unit.

The signal assignments are summarised in Table 1 and shown in the edited HSQC spectrum with abbreviations (Figs 4A,B and S1).

**Table 1:** NMR signal assignments of LCC, lignin and polysaccharide as inferred through ^1^H–^13^C correlation spectroscopy.

Labels	δ_H_ (ppm)	δ_C_ (ppm)	Assignments
α	4.50	80.2	LCC C_α_–H_α_ in β-*O*-4′″ substructures
β	4.30	83.0	LCC C_β_–H_β_ in β-*O*-4′″ substructures linked to guaiacyl units
γ_1_, γ_2_	3.32, 3.60	60.1	LCC C_γ_–H_γ1_, H_γ2_ in β-*O*-4′″ substructures
G_2_	6.98	111.2	LCC C_2_–H_2_ in guaiacyl units
G_6_	6.75	120.4	LCC C_6_–H_6_ in guaiacyl units
1′(1″), 1′	4.54, 4.46	100.5, 100.6	LCC C_1_–H_1_ in β- d -mannopyranoside (a part of glucomannan)
2′	3.65	70.5	LCC C_2_–H_2_ in β- d-mannopyranoside (a part of glucomannan)
3′	3.62	71.0	LCC C_3_–H_3_ in β- d-mannopyranoside (a part of glucomannan)
4′, 4′	3.68, 3.76	77.6, 78.0	LCC C_4_–H_4_ in β- d-mannopyranoside (1″ → 4′-linked mannose units)
5′	3.64	69.0	LCC C_5_–H_5_ in β- d-mannopyranoside (a part of glucomannan)
6′	3.48	68.1	LCC C_6_–H_6_ in β- d-mannopyranoside (a part of glucomannan), (center of width in signals)
A_α(G,t)_	4.72	71.4	C_α_–H_α_ in β-*O*-4′″ substructures (*threo* form)
A_α(G,e)_	4.72	70.9	C_α_–H_α_ in β-*O*-4′″ substructures (*erythro* form)
A_β(G,e)_	4.23	84.3	C_β_–H_β_ in β-*O*-4′″ substructures linked to a guaiacyl unit (*erythro* form)
A_β(G,t)_	4.27	83.6	C_β_–H_β_ in β-*O*-4′″ substructures linked to a guaiacyl unit (*threo* form)
A_γ1,_ A_γ2_,	3.22, 3.57	59.9	C_γ_–H_γ1_, H_γ2_ in β-*O*-4′″ substructures
G_2_	6.97	111.3	C_2_–H_2_ in guaiacyl units (center of width in signals)
G_5_	6.93	115.1	C_5_–H_5_ in guaiacyl units (center of width in signals)
G_6_	6.80	119.0	C_6_–H_6_ in guaiacyl units (center of width in signals)

Abbreviations:

LCC, α-ether (benzyl ether)-type lignin–carbohydrate complex; G, guaiacyl lignin units; Man, mannose units; A, β-*O*-4′″ substructures; *e, erythro* form; *t, threo* form; underline, signals derived by those structural diversity. Corresponding chemical structures with lebels are shown in Fig. 3.

### Assignment of α-ether-type LCC through-bond NMR correlations in a lignin moiety

**Figure 5:**
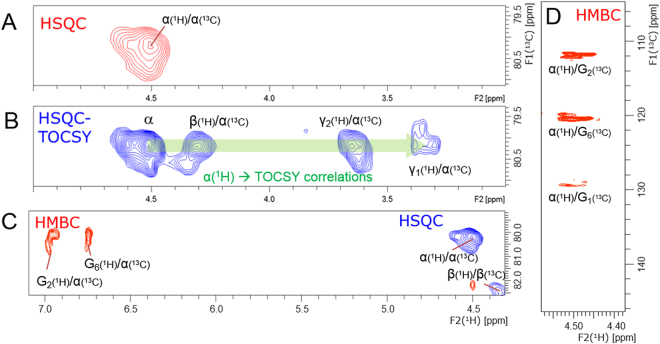
Partial 2D-NMR spectra of one-bond and long-range correlations around the β-*O*-4′″ lignin subunits of the α-ether-type LCC. (**A**) 2D ^1^H–^13^C HSQC NMR spectrum of the α-position of β-*O*-4′″ lignin subunits at δ_H_/δ_C_ = 4.50 ppm/80.2 ppm. (**B**) ^1^H–^13^C HSQC–TOCSY NMR spectrum of the α-carbon of the β-*O*-4′″ lignin subunits at δ_C_ = 80.2 ppm displaying the TOCSY correlation between the α-proton and the β-, γ_1_- and γ_2_-protons of the β-*O*-4′″ lignin subunits. (**C**) ^1^H–^13^C HSQC (blue) and ^1^H–^13^C HMBC (red) overlaid NMR spectra showing the correlations of the α-carbon of the β-*O*-4′″ lignin subunits (δ_C_ = 80.2 ppm) with the G_2_ and G_6_ protons of the guaiacyl lignin units. (**D**) ^1^H–^13^C HMBC NMR spectrum, showing the correlations of the α-proton of the β-*O*-4′″ lignin subunits (δ_H_ = 4.5 ppm) with the G_2_, G_6_ and G_1_ carbons of the guaiacyl lignin units.

Signal assignment started from the Cα–Hα correlation signal of the α-ether-type LCC observed at δ_H_/δ_C_ = 4.50 ppm/80.2 ppm in HSQC (Fig. 5A). TOCSY correlations between Hα and β, γ_2_ and γ_1_ protons in the guaiacyl lignin were observed at δ_H_ = 4.30 ppm, 3.60 ppm, and 3.32 ppm, respectively, in 2D HSQC-TOCSY (Fig. 5B). HMBC correlations between Cα at δ_C_ = 80.2 ppm and G_2_ and G_6_ protons in the guaiacyl lignin units were observed at δ_H_ = 6.95 and 6.74, respectively (Fig. 5C). HMBC correlations between Hα and G_2_, G_6_ and G_1_ carbons in the guaiacyl lignin unit were observed at δ_C_ = 111.2 ppm, 120.4 ppm, and 129.4 ppm, respectively (Fig. 5D).

**Figure 6:**
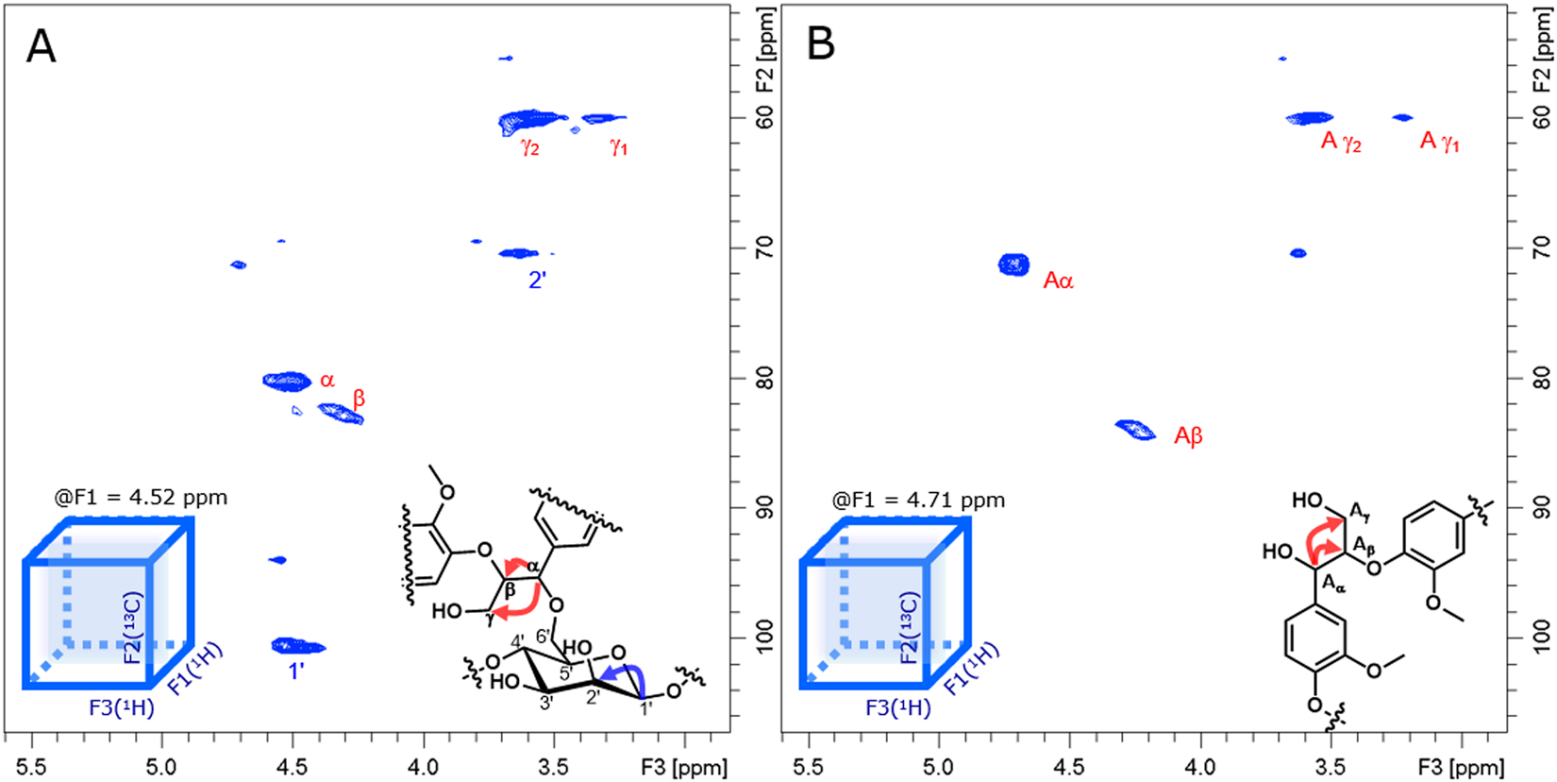
3D ^1^H–^13^C TOCSY-HSQC NMR spectra, showing the correlations of the α-proton of the β-*O*-4′″ lignin subunits with the β-, γ_1_- and γ_2_-protons of the guaiacyl lignin unit in the α-ether-type LCC (**A**) and of the free β-*O*-4′″ lignin unit (**B**) in the LCC fraction. Arrows in the structural formulae indicate observed couplings in each spectrum. (**A**) A slice of the F2–F3 plane of ^1^H–^13^C (TOCSY-HSQC at ^1^H shift of δ_H_ = 4.52 ppm), showing the α proton correlated to the β-, γ_1_- and γ_2_-protons of the lignin unit in the α-ether-linked LCC. Since the chemical shift of the α proton accidentally coincides with that of the C-1′ proton, also shown is the correlation of the C-1′ proton with the C-2′ proton of the β-d-mannose unit in the α-ether-type LCC. (**B**) A slice of the F2–F3 plane of ^1^H–^13^C (HSQC-TOCSY at ^1^H shift of δ_H_ = 4.71 ppm), showing the correlation of the α proton to the β-, γ_1_- and γ_2_-protons of the free β-*O*-4′″ lignin units (uncombined with carbohydrates).

In the F2–F3 plane (^13^C–^1^H plane) of 3D TOCSY-HSQC were observed correlations at ^1^H shift δ_H_ = 4.52 ppm, β-, γ_1_- and γ_2_- (Fig. 6A), which can be distinguished from those of the free β-*O*-4′″ lignin units (uncombined with carbohydrates; see Fig. 6B). The signals in the spectrum reported in Fig. 6A clearly indicate the existence of correlations of the phenylpropane unit in the α-ether-type LCC.

### Assignment of α-ether-type LCC through-bond NMR correlations between the lignin and carbohydrate moieties

**Figure 7:**
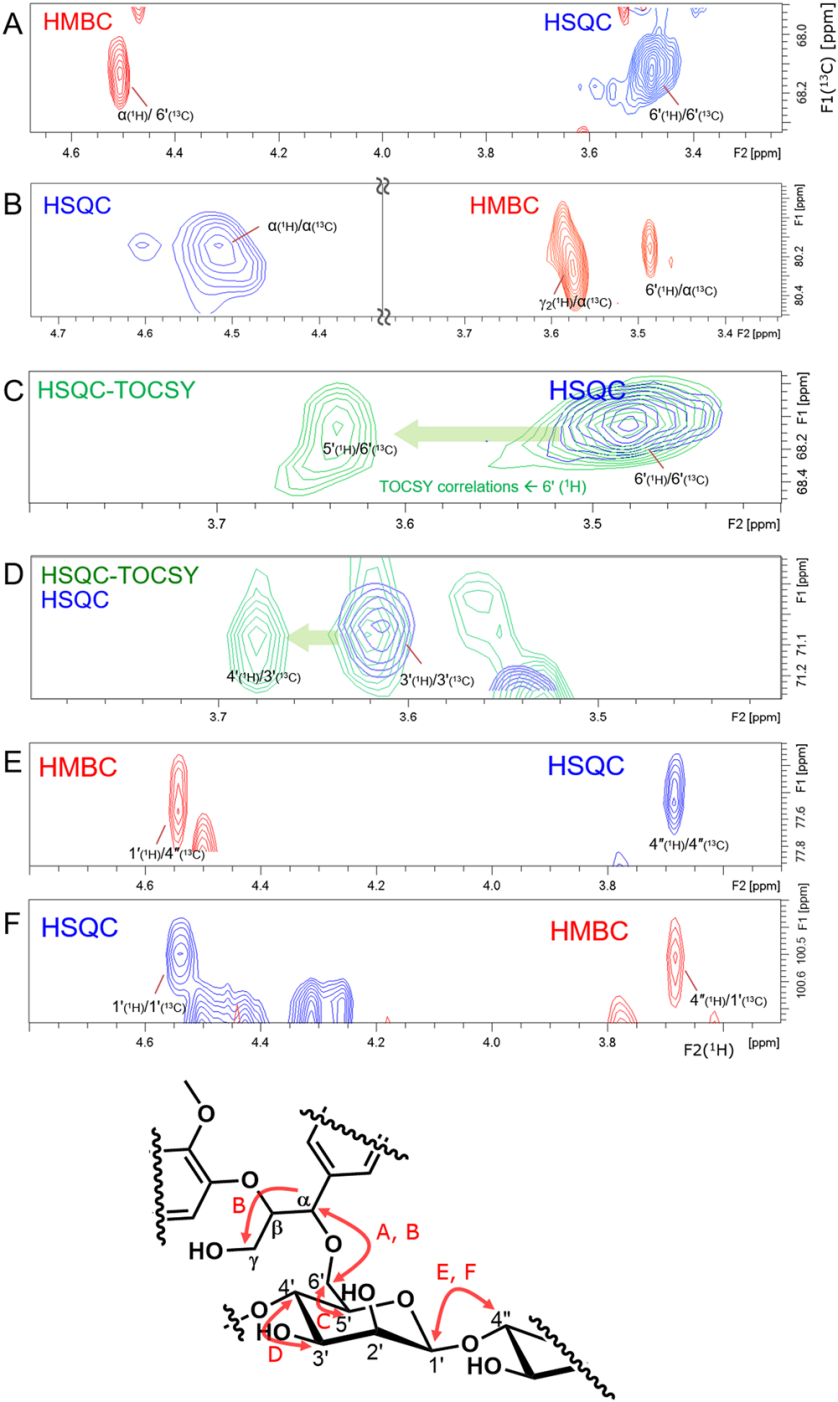
Partial 2D-NMR spectra, showing correlations between the LC linkage and the carbohydrate moiety. Arrows in the structural formula indicate observed couplings in each spectrum. (**A**) ^1^H–^13^C HSQC (blue) and ^1^H–^13^C HMBC (red) NMR spectra. The α C-H of the β-*O*-4′″ lignin subunits, δ_H_/δ_C_ = 4.50 ppm/80.2 ppm in HSQC, correlates with the C-6′ proton through the ether linkage. A correlation with the γ_2_ proton of the guaiacyl unit is also observed. (**B**) ^1^H–^13^C HSQC (blue) and ^1^H–^13^C HMBC (red) overlaid NMR spectra. C-6′, δ_H_/δ_C_ = 3.48 ppm/68.1 ppm, in HSQC correlates with the α proton of the β-*O*-4′″ lignin subunit through the ether linkage. (**C**) ^1^H–^13^C HSQC (blue) and 2D ^1^H–^13^C HSQC–TOCSY(green) overlaid NMR spectra. C-6′ correlates with the 5′ proton at δ_H_ = 3.64 ppm. (**D**) ^1^H–^13^C HSQC (blue) and 2D ^1^H–^13^C HSQC–TOCSY(green) overlaid NMR spectra. The C-3′ carbon correlates with the 4′ proton at δ_H_ = 3.68 ppm. (**E,F**) ^1^H–^13^C HSQC (blue) and ^1^H–^13^C HMBC (red) overlaid NMR spectra. The HMBC correlation from the C-4′ carbon to the 1″ proton (**E**) and the HMBC correlation from the anomeric C-1″ carbon to the 4′ proton (**F**) are observed. These correlations reveal β-1,4-linked mannosyl residues of the α-ether-type LCC.

The dominant correlation signal observed in the HMBC was the one due to the correlation between the α-carbon at δ_C_ = 80.2 ppm and the carbohydrate proton at δ_H_ = 3.48 ppm, as shown in Fig. 7A. Symmetrically, a correlation was observed in the HMBC between the α-proton at δ_H_ = 4.50 ppm and the carbohydrate carbon at δ_C_ = 68.1 ppm, as shown in Fig. 7B. The HSQC correlation peak of δ_H_/δ_C_ = 3.48 ppm/68.1 ppm is assigned to C*-*6′ through the multiplicity-edited HSQC experiment, in which signals originating from CH and CH_3,_ and those from CH_2_ are observed in opposite sign, a test that demonstrated that the signal of C*-*6′ at δ_H_/δ_C_ = 3.48 ppm/68.1 ppm was due to the methylene group (Fig. 4A,B).

### Assignment of α-ether-type LCC through-bond NMR correlations in the carbohydrate moiety

Carbohydrate correlations within the LCC were identified in the 2D HMBC, 2D HSQC-TOCSY and 3D TOCSY-HSQC spectra. A correlation signal due to the C*-*6′ carbon at δ_C_ = 68.1 ppm and the C*-*5′ proton at δ_H_ = 3.64 ppm was observed in the 2D HSQC-TOCSY (Fig. 7C).

**Figure 8:**
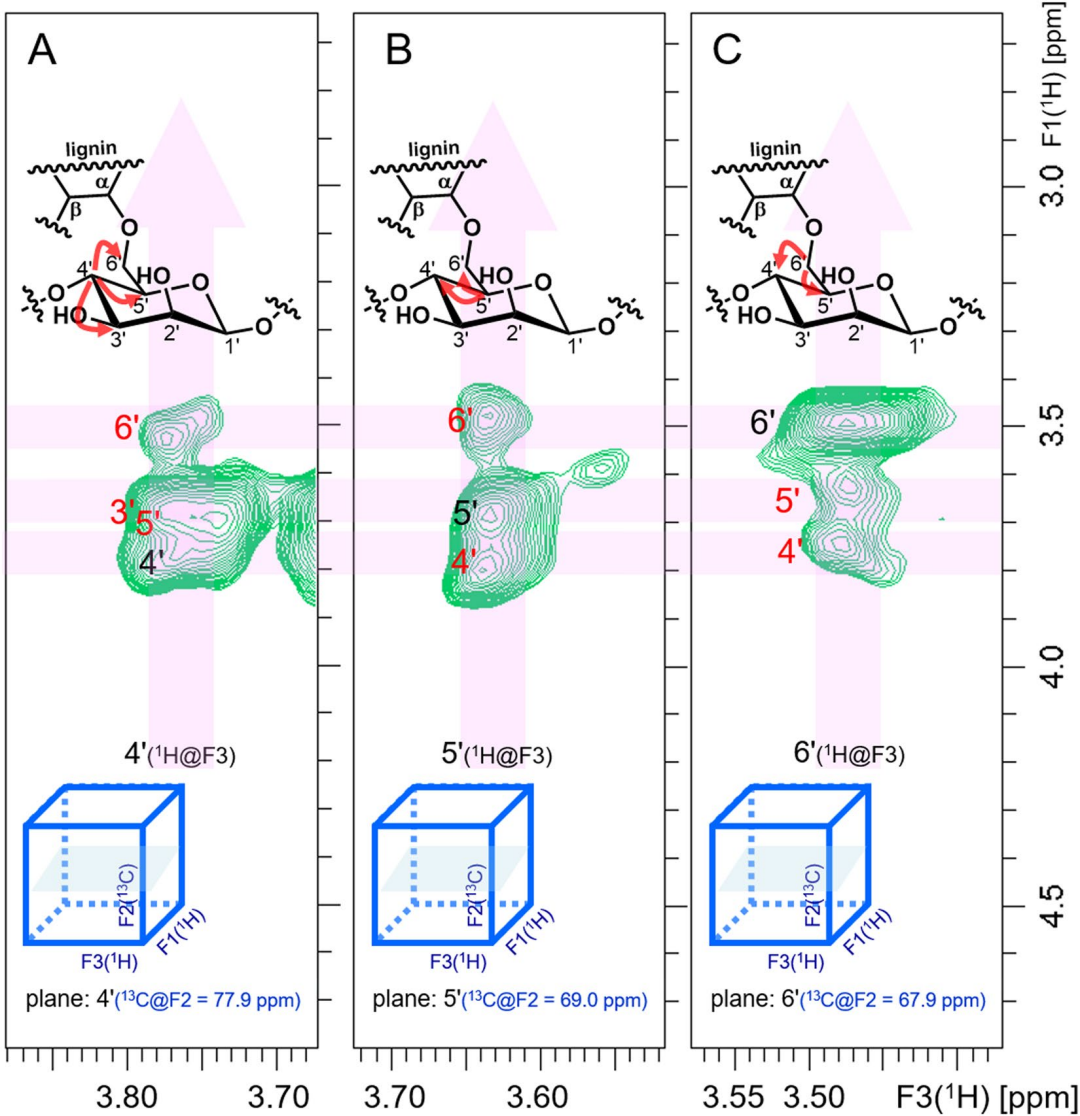
3D TOCSY-HSQC NMR spectrum of the α-ether-type LCC. Arrows in the structural formulae indicate observed couplings in each spectrum. (**A**) A slice of the F1–F3 plane of ^1^H–^1^H (TOCSY-HSQC at a ^13^C shift, δ_C_ = 77.9 ppm), showing the carbohydrate correlations of the α-ether-type LCC: C-4′ proton correlated to the C-5′ proton followed by the C-6′ proton of the β-d-mannose unit in the α-ether-type LCC. Correlation to the C-3′ proton is overwrapped with that to the C-5′ proton. (**B**) A slice of the F1–F3 plane of ^1^H–^1^H (TOCSY-HSQC at ^13^C shift of δ_C_ = 68.9 ppm), showing the carbohydrate correlations of the α-ether-type LCC: the C-5′ proton is correlated to the C-4′ and C-6′ protons of the β-d-mannose unit in the α-ether-type LCC. (**C**) A slice of the F1–F3 plane of ^1^H–^1^H (TOCSY-HSQC at ^13^C shift of δ_C_ = 67.9 ppm), showing the carbohydrate correlations of the α-ether-type LCC: the C-6′proton is correlated to the C-5′ proton followed by C-4′ proton of the β-d-mannose unit in the α-ether-type LCC.

TOCSY correlations between C*-*6′ and C-3′ were observed in the 3D TOCSY-HSQC (Fig. 8). In the slices of the F1–F3 planes, ^1^H–^1^H correlations were observed from C-4′ (δ_H_/δ_C_ = 3.68 ppm/77.6 ppm), C-5′, C-3′ (overlapped with 5′) and C-6′ (Fig. 8A); correlations from C-5′ (δ_H_/δ_C_ = 3.64 ppm/69.0 ppm) to C-4′ and C-6′ (Fig. 8B) and from C-6′ (δ_H_/δ_C_ = 3.48 ppm/68.0 ppm) to C-5′ and C-4′ (Fig. 8C) were also observed. Furthermore, TOCSY correlations between C-3′ and C-4′ were observed in the 2D HSQC-TOCSY (Fig. 7D).

A three-bond HMBC correlation was observed that started from a C-4′ carbon at δ_C_ = 77.6 ppm and reached a C-1″ proton at δ_H_ = 4.54 ppm(Fig. 7E). A symmetric 3-bond HMBC correlation from the C-1″ carbon at δ_C_ = 100.5 ppm to the C-4′ proton at δ_H_ = 3.68 ppm was observed (Fig. 7F). These correlations demonstrate the presence of the 1–4 glycosidic linkage of the polysaccharide. Additionally, a TOCSY correlation from C-1′ to C-2′ was observed in 3D TOCSY-HSQC, a slice of the F2–F3 plane at a ^1^H shift of δ_H_ = 4.5 ppm, as shown in Fig. 6A.

TOCSY correlations among C-3′, C-4′, C-5′ and C-6′ via spin–spin couplings were observed, whereas correlations among C-2′, C-3′ and C-1′ were not. In general, the *J* coupling between axial and equatorial protons is weak, and the sequential axial-to-axial proton TOCSY correlations were clearly observed. Evidence thus indicates that the configuration of the C-2′proton is equatorial, resulting in the missing correlation signal through C-2′. These results support the idea that the carbohydrate moiety is d-mannose, a C-2 epimer of d-glucose.

**Figure 9:**
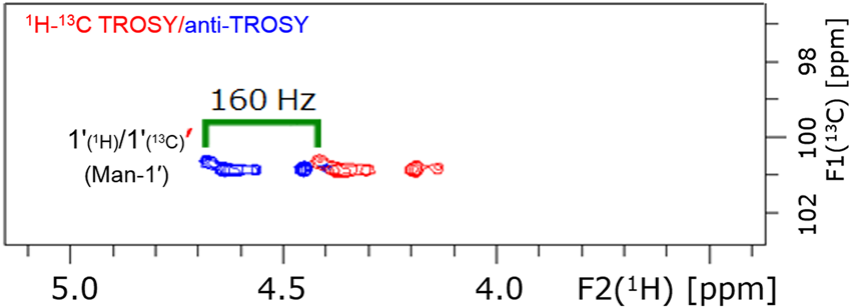
Overlaid, selectively decoupled ^1^H–^13^C TROSY and anti-TROSY NMR spectra of fractionated LCC fragments. The coupling constant ^1^*J*_CH_ is labelled in the figure. A ^1^*J*_CH_ value of 160 Hz at C*-*1′ of the α-ether-type LCC indicates a β-anomeric sugar configuration.

The anomeric configuration of the sugar moiety was determined by the coupling constant, ^1^*J*_CH_, of C-1′. The accurate value of the coupling constant was measured by the chemical shift difference between the correlation peaks in the TROSY and anti-TROSY experiments. A value of 160 Hz was thus determined for ^1^*J*_CH_, indicating a β-anomeric sugar configuration (Fig. 9).

From these findings, we determined that natural LCC has an α ether LC bond at the C-6′ position of *β-*1,4-linked-d-mannose in glucomannan.

## Discussion

Although LCCs have been studied for more than a century, direct evidence of the presence of chemical bonds between lignin and carbohydrates has not yet been obtained. We have succeeded in purifying an LCC fraction to a level superior to that achieved by conventional preparation methods to convincingly prove the existence of a direct lignin–carbohydrate covalent bond. Correlations observed in 2D and 3D NMR spectra, including ^1^H–^13^C three-bond correlations between lignin and carbohydrates, demonstrated the presence of α-ether-type LCCs. We have assigned correlations among atoms directly involved in the lignin–carbohydrate linkage and those located in its vicinity, including lignin aromatics, lignin interunit aliphatics and hexose of C1 to C6.

Difficulties in elucidating the structure of LCC arise from the fractionation of LCCs. LC linkages in natural wood cell walls are present in low frequency and diverse throughout macromolecules. In this study, we used water-soluble neutral LCCs from softwood obtained by water extraction of the residue of milled wood lignin (MWL). These LCCs contain pendant-like lignin, that is relatively small lignin molecules attached to hemicellulose chains as ‘pendants’. These compounds are suitable for LC bond analysis due to the relatively high frequency of occurrence of the LC bond and the simplicity of the structure of the hemicelluloses. Another challenge in LC bond analysis is the detection and assignment of the linkages between lignin and carbohydrates. NMR spectroscopy is a powerful tool for the structural analysis of lignocelluloses; however, detection and correlation analysis of LC linkages has proven difficult due to the low number of such linkages. In particular, the low number of LC bonds and the low sensitivity of the spectroscopic method when investigating macromolecules with short T2 values render the observation of long-range correlation signals between lignin and carbohydrates challenging to achieve. We applied glycohydrolase treatment and separated LCC fragments from the hydrolysates using a polyvinyl gel having an affinity to lignin and displaying a size-exclusion effect.

The present study provides the first direct evidence for an α ether bond between lignin and hemicellulose. Thus far, NMR has been applied to the analysis of lignin-carbohydrate bonds, but the previous methods depended on estimation of the structure by chemical shift correlation between model compounds and extracted LCC samples. However, the chemical shifts of these signals are not exactly identical. Also, overlapping signals from other unidentified structures cannot be excluded. In this study, we first observed the long-range correlations through an α-ether bond between lignin and the primary hydroxyl group of a mannose residue in glucomannan, and identified lignin–carbohydrate bonds by complete connectivity analysis from the phenylpropane unit of lignin to the carbohydrate moiety in hemicellulose.

This study strongly supports the idea that the linkage is formed during the polymerisation of lignin in the presence of hemicelluloses via quinone methide formation and subsequent nucleophilic attack of the primary hydroxyl group of the hemicelluloses as found in the experiments that α-ether bonds were preferentially formed between lignin and C-6 position of mannose residue by peroxidase-catalysed dehydrogenative polymerization of coniferyl alcohol in the presence of glucomannan^30^. The methodologies we devised to conduct this study can be applied to various LCC fractions from different plant sources to obtain detailed structural information on LCCs in plant cell walls.

## Conclusions

Determining the possible presence of covalent bonds linking lignin and carbohydrates has been an important research goal in plant science for over 150 years. Although abundant supporting evidence for the existence of these chemical bonds has been accumulated, no direct proof has been reported for these interactions. To demonstrate the existence of these bonds, we purified LCC fragments from a neutral LCC fraction using mannanase and a polyvinyl gel having an affinity to lignin and exerting a size-exclusion effect. We applied HMBC and ^13^C-edited TOCSY-HSQC analyses to the purified LCC fragments and conclusively detected correlation signals between the C-6 position of the β-mannose residue in glucomannan and the α position of lignin, indicating the presence of an α ether bond between these species. This LCC study provides deeper understanding of plant cell-wall structures and the roles of LC bonds in the physical properties of cell walls, a key research objective for developing conversion technologies for plant biomass through component separation.

## Methods

### Sample preparation

Fractions of LCCs and MWL were prepared from the reaction wood of Japanese red pine (*Pinus densiflora* Zieb. et Zucc.) collected in Kyoto, Japan. The pine wood was extracted with ethyl alcohol–benzene mixture (1:2, v/v) and 0.25% aqueous potassium acetate^27^. The wood meal was dried and subsequently vibromilled for 48 h under a nitrogen atmosphere with external cooling by tap water. The crude MWL fraction was extracted twice from the wood meal with 80% aqueous dioxane for 48 h at room temperature. LCCs were extracted from the wood residue with cold water (20 °C) for 12 h, and then with hot water (80 °C) for 5 h. The two extracts were combined and concentrated to a small volume. A water-soluble LCC (LCC-WE) fraction was precipitated from the concentrated solution by addition of five times the volume of ethyl alcohol. LCC-WE was then fractionated into neutral (C-1-M), acidic (C-l-A) and lignin-rich (C-1-R) sub-fractions by anion-exchange chromatography on DEAE-Sephadex A-50 (CO_3_^2−^ form), as described previously^27^. The neutral fraction (C-1-M) was used in this study. A flowchart of this sample preparation procedure is presented in Fig. 2A. Smith degradation of C-1-M was conducted by reaction with sodium periodate at 4 °C for 20 days followed by reduction with NaBH_4_ and hydrolysis with TFA at 100 °C for 3 h. The products were analysed by gas chromatography using a Shimadzu GC-17 on an Ulbon HR-SS-10 column (25 m × 0.25 mm i.d., Shinwa Chemical Industries Ltd, Kyoto) with a temperature gradient from 100 °C to 210 °C at a rate of 5 °C/min.

### Enzymatic digestion and purification of LCC fragments

The C-1-M fraction (8.0 g) was digested at 45 °C for 24 h in 0.1 M sodium acetate buffer (pH 4.5) by *Cellulosin* GM5 (HBI Enzymes Inc., Hyogo, Japan), which displays mainly mannanase activity. *Cellulosin* GM5 mannanase activity was 1786 U, when digesting glucomannan from Konjac (Wako Pure Chemical Industries, Ltd., Osaka, Japan). 1U is defined as the amount of enzyme releasing 1μmol of reducing sugar per min. After the digestion, LC fragments were separated using a size-exclusion polyvinyl gel with an affinity for lignin^13^. The enzymatic hydrolysates were loaded onto the hydrophobic interaction/size exclusion gel TOYOPEARL HW50 (80 cm, 8 cm i.d., Tosoh Bioscience LLC, Tokyo, Japan) and eluted with water to wash out the carbohydrate fragments. The eluent was then converted to 50% aqueous 1,4-dioxane to recover the adsorbed LCC fragments. The eluate was collected, concentrated with a rotary evaporator, and purified by HPLC (HITACHI L7000 DAD system, Tokyo, Japan). The conditions for HPLC were as follows: mobile phase, water and 1,4-dioxane, analytical column, TOYOPEARL HW50 (30 cm, 2 cm i.d.), detection wavelength of 190–400 nm, flow rate of 3.0 mL/min, and gradient of 0% to 50% increase in 1,4-dioxane over 10–50 min. The HPLC eluates were collected between 12 and 32 min and analysed by NMR. The flow chart of this procedure and schematic description of it are reported in Fig. 2A,B, respectively.

### NMR experiments

Samples were dissolved in 0.5 mL of DMSO-*d*_6_ and transferred to an NMR tube. NMR spectra were recorded at a temperature of 313 K using Bruker AVANCE 600 MHz spectrometers equipped with a 5-mm cryoprobe. A 1D ^1^H NMR spectrum of the sample was acquired using an acquisition time (AQ) of 0.9 s and an inter scan delay (D1) of 2.0 s; 32 scans were recorded. The HSQC spectrum was recorded by performing an adiabatic HSQC experiment (Bruker pulse program ‘hsqccetgpsisp2.2′) with the following parameters; AQ, 0.10 s, D1, 2.0 s, the spectral window of 16 ppm in F2 and 165 ppm in F1 with 2048 × 512 increments, and 32 scans per increment. The ^1^*J*_CH_ used was 145 Hz. Prior to Fourier transformation, the data matrices were zero filled to 1024 points in the ^13^C dimension and processed with a Gaussian window function in the F2 dimension and a sine-bell square window function in the F1 dimension. The multiplicity-edited HSQC was recorded by the Bruker pulse program ‘hsqcedetgp’. The ^1^H-^13^C HMBC spectrum was recorded by performing a magnitude-mode ge-2D HMBC using low-pass *J*-filter (Bruker pulse program ‘hmbcgplpndqf’) with the following parameters; AQ, 0.12 s, D1, 1.2 s, spectral window of 14 ppm in F2 and 150 ppm in F1 with 2048 × 512 increments, 48 scans per increment and processed with a sine-bell window function in both F1 and F2 dimensions. The 2D ^1^H-^13^C HSQC-TOCSY spectrum was recorded by the Bruker pulse program ‘hsqcdietgpsi’ with the following parameters; AQ, 0.14 s, D1, 2.0 s, spectral window of 11 ppm in F2 and 130 ppm in F1 with 2048 × 512 increments; 32 scans per increment, 100 ms of TOCSY spin lock period, and processed with a sine-bell square window function in both F1 and F2 dimensions. The 3D ^1^H-^13^C TOCSY-HSQC spectrum was recorded by the Bruker pulse program ‘mlevhsqcetgp3d’ with the following parameters; AQ, 0.07 s, D1, 1.0 s, spectral window of 12 ppm in F3, 58 ppm in F2 and 5.5 ppm in F1 with 1024 × 384 × 64 increments, 8 scans per increment, 100 ms of TOCSY spin lock period, and processed with a sine-bell square window function in all dimensions. Data processing was performed using standard Bruker Topspin-NMR software (ver. 3.1). The solvent (DMSO) peak was used as an internal chemical shift reference point (δ_H_/δ_C_ = 2.49 ppm/39.6 ppm). The^1^H-^13^C TROSY spectrum was recorded by performing an experiment modified from pulse program ‘trosyetgpsisp’. One-bond coupling constants ^1^*J*_CH_ are measured by a selection of a pair of single multiplet components in TROSY.

## Electronic supplementary material

Supplementary Information
