# Supplementary material for: Direct evidence for α ether linkage between lignin and carbohydrates in wood cell walls
Source: Sci Rep. 2018 Apr 25;8:6538. doi: 10.1038/s41598-018-24328-9 (PMC5916878; doi:10.1038/s41598-018-24328-9)
Supplement: Supplementary file 1 — Supplementary Information [file 41598_2018_24328_MOESM1_ESM.docx]

**Supplementary Information**

**Direct evidence for α ether linkage between lignin and carbohydrates in wood cell walls**

Hiroshi Nishimura^1^, Akihiro Kamiya^2^, Takashi Nagata^2^, Masato Katahira^2^ & Takashi Watanabe^1^*

**Affiliations:**

^1^Research Institute for Sustainable Humanosphere (RISH), Kyoto University, Uji 611-0011, Japan.

^2^Institute of Advanced Energy (IAE), Kyoto University, Uji 611-0011, Japan.

*twatanab@rish.kyoto-u.ac.jp


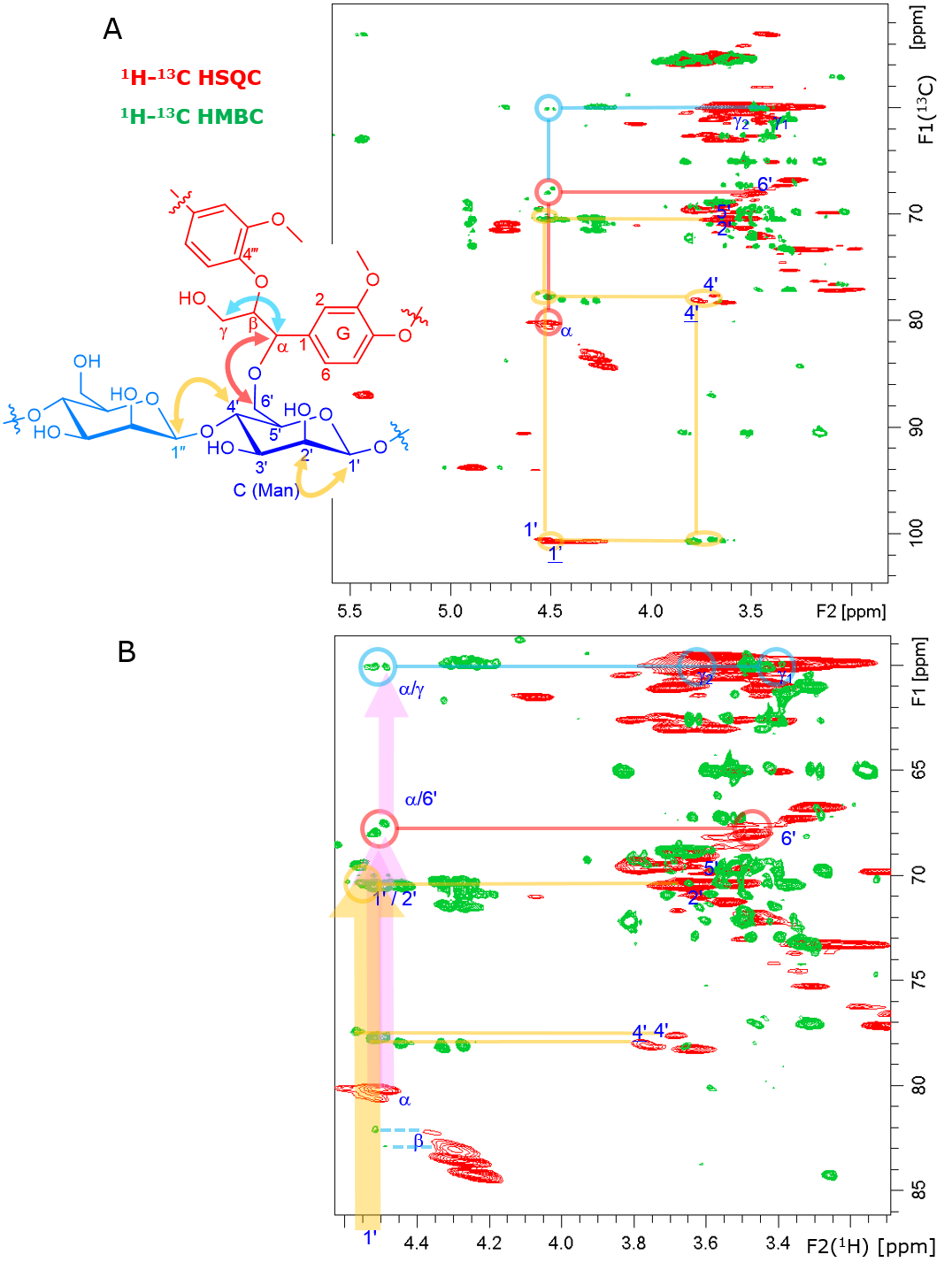


**Figure S1.** Overlaid ^1^H–^13^C HSQC (red) and ^1^H–^13^C HMBC (green) 2D-NMR spectra of LC linkages and carbohydrate correlations around the α-ether-type LCC; overall (**S1A**) and magnified (**S1B**) spectra. The C-6′, δ_H_/δ_C_ = 3.48 ppm/68.1 ppm in HSQC, correlates with the α proton of the β-*O*-4‴ lignin subunit in the α-ether-type LCC, δ_H_/δ_C_ = 4.50 ppm/80.2 ppm, through the ether linkage (shown in red-coloured lines). ^1^H–^13^C HMBC correlations between the α proton and the γ_1_- and γ_2_- carbons of the guaiacyl lignin unit (shown in blue-coloured lines) and those between the C-1′(1″) proton and the C-4′ and C-2′ carbons (shown in yellow-coloured lines in S1B) are highlighted. Arrows in the structural formula indicate selected couplings in each spectrum.
